# Descriptive Cross-Sectional Study on Knowledge, Awareness, and Adherence to Medication among Hypertensive Patients at a Tertiary Care Centre in Colombo District, Sri Lanka

**DOI:** 10.1155/2020/1320109

**Published:** 2020-08-03

**Authors:** S. Pirasath, A. G. H. Sugathapala, K. Wanigasuriya

**Affiliations:** ^1^Colombo South Teaching Hospital, Kalubowila, Sri Lanka; ^2^Faculty of Medical Sciences, University of Sri Jayewardenepura, Nugegoda, Sri Lanka

## Abstract

**Objective:**

This study was aimed to assess the patient's knowledge and awareness about hypertension and adherence to antihypertensive medication among hypertensive patients.

**Methods:**

The descriptive cross-sectional study was conducted in three medical clinics of Colombo South Teaching hospital, Kalubowila, Sri Lanka, from April 2019 to September 2019. Total of 384 hypertensive patients were recruited by systematic randomized controlled sampling and interviewed with validated questionnaires to assess their knowledge about hypertension and adherence to antihypertensive medication. Data were analyzed using SPSS (version 21) analytical package, and the chi-squared test was performed.

**Results:**

The total sample consisted of 384 hypertensive patients with a mean age of 59.32 (±12.34SD). This included 180 (46.9%) males and 204 (53.1%) females. The male : female ratio was approximately 9 : 10. Most of patients were with primary and ordinary educational status (65.9%), normal body mass index (54.9%), mild elevation of LDL cholesterol (76.3%), and coexistent ischemic heart disease (39.6%). The knowledge about hypertension among majority of patients was reasonable. However, they were unaware about normal values of blood pressure (69%, 95% of CI 1.92–2.09) and diagnostic values of hypertension (90.1%, 95% of CI 2.26–2.40). Moreover, they were unaware of their blood pressure values at time of diagnosis (75.3%, 95% of CI 2.09–2.25), at recent clinic visit (71.3%, 95% of CI 2.0-2.17), and target level (81.8%, 95% of CI 2.25-2.41). Most patients had adequate knowledge about the risk factors and complications of hypertension and were aware of their target organ damage (70.3%). Most patients believed that medication alone is not sufficient to control blood pressure (41.7%, 95% of CI 1.40-1.51) and adequate control of their blood pressure reduces complications (68.2%, 95% of CI 1.37-1.51). Most of the patients (71.8%) had reasonable good drug compliance. The forgetfulness was commonly attributed for nonadherence (69%, 95% of CI 1.26-1.36).

**Conclusions:**

The knowledge about hypertension among majority of patients was reasonable. But, they were unaware about their disease status and their diagnosis, target, and recent blood pressure values. Most of patients had adequate knowledge about the risk factors and complications of hypertension. However, they were unaware about their target organ damage due to hypertension. The drug compliance was reasonable among them. The forgetfulness was common reason for nonadherence. Therefore, healthcare professionals should implement individualized educational programmes to increase the awareness of disease status, appropriate blood pressure levels, and adherence of treatment to improve the outcome of patients.

## 1. Introduction

Hypertension is a common noncommunicable disease and one of the major risk factors for stroke, coronary artery diseases, and chronic kidney disease [1]. Hypertension is a significant global burden among noncommunicable diseases which is a public health problem strongly related to the urbanization and socioeconomic changes favoring sedentary life style [2]. Effective health services are needed to control hypertension in the world because it has a major economic impact ranging from medical costs to human capital loss and decrease in productivity [3].

Prompt recognition of the importance of systolic blood pressure is mandatory for medical professionals as one of the major public health and medical challenges in the prevention and treatment of hypertension [4]. Screening for elevated systolic blood pressure (SBP) has been identified as an important medical challenge in the prevention and treatment of hypertension. The lack of knowledge and awareness on hypertension and its complications among public is a major barrier to diagnose hypertension. Patient education is a key component in the programs and interventions designed to control hypertension, so it is therefore important to assess the patients' knowledge and awareness of hypertension. Efforts to control hypertension have included improving public knowledge and awareness on the risks and complications of hypertension. The good knowledge, awareness, and attitudes of hypertension among hypertensive population will lead to good control and reduce the complications of hypertension. This study was aimed to assess knowledge, awareness of hypertension, and adherence to medication among hypertensive patients in a tertiary care centre of western Sri Lanka.

## 2. Methods

### 2.1. Study Design, Period, and Participants

A cross-sectional study was conducted among hypertensive patients attending medical clinics, Colombo South Teaching Hospital, Kalubowila, from 1^st^ April, 2019, to 30^th^ September, 2019.

### 2.2. Sample Size and Sampling Technique

There were no previous studies carried out to evaluate the knowledge, awareness of hypertension, and adherence to medication among hypertensive patients attending medical clinics, Colombo South Teaching hospital, Kalubowila, Colombo. Therefore, sample size was calculated by a single population proportion formula considering 5% of absolute precision required on either side of the proportion, 0.5 of anticipated population proportion, and critical value (1.96) with specified confidence interval of 95%. With the addition of 5% nonresponse rate, the yield sample size was 384. The number of potentially eligible participants and who refused were 9649 and 24, respectively. The participants were selected by simple random sampling technique.

Sample size calculation is as follows:

(1) $$\begin{array}{c} N=\frac{Z2p\left(1-p\right)}{d2}, \end{array}$$

where *N* is the minimal sample size, *Z* is the critical value (1.96) of specified confidence interval which is 95%, *p* is the anticipated population proportion (0.5), *d* is the absolute precision required on either side of the proportion (5%), and *N* = 384.

Therefore, the calculated minimum sample size according to the above equation is as follows.

A nonresponse rate of 5% is assumed. Thus, the final sample = 384.

The patients above 18 years who are mentally competent, previously diagnosed as hypertensive by consultant physicians, and attending to medical clinics for 3 months or more at Colombo South Teaching Hospital, Kalubowila, were included and those who were pregnant and those who were unable to give consent was excluded in this study.

### 2.3. Data Collection and Study Instruments

The pretested validated questionnaire was used to collect the data of knowledge, awareness of hypertension, and adherence to medication among the hypertensive patients. The variables which were studied in this research were sociodemographic variables, knowledge and awareness of hypertension, adherence to medications, and reasons for nonadherence.

#### 2.3.1. Numerical Variables

The blood pressure measurements were measured by medical officers on day of interview, using a mercury sphygmomanometer, in a comfortable resting position, sitting with forearm supported and the palm upward. The target blood pressure of <130/80 mm Hg for patients with diabetes and chronic kidney disease and <140/90 mm Hg for patients with nondiabetic and nonchronic kidney disease (7) was considered as target levels. The weight and height were measured using a standard electronic weight scale and standard height scale, and BMI was calculated based on weight and height using standard equation (BMI = weight (kg)/height (m^2^)). The cholesterol values were recorded from their clinic records.

#### 2.3.2. Study Instruments

*(1). Interviewer-Administrated Pretested Questionnaires*. The Hypertension Fact Questionnaire was designed as a tool, using the existing literature, practicing physicians, and cardiologists to assess the knowledge and awareness among the hypertensive patients. It was a pretested validated questionnaire. It was a self-prepared pretested questionnaire based on preexisting facts related to hypertension without copying originality and was obtained content expert validation of facts from two medical professors, two consultant physicians, one cardiologists, two senior registrars, and one highly qualified English, Sinhala, and Tamil teachers. It was pretested during the pilot study with patients. The questionnaire was initially designed in English and then translated to Sinhala and Tamil languages. The questionnaire consists of twenty-one and twelve questions with different appropriate responses to assess the patients' knowledge and awareness on hypertension, respectively. The knowledge and awareness of hypertension among participants were calculated depending on the number of questions with the correct responses of each participant. Their medication adherence and the reasons for nonadherence were studied using the modified version based on the Morisky Medication Adherence Scale.

### 2.4. Data Analysis and Processing

The collected data were entered in Microsoft Excel sheet and were analyzed using SPSS (version 18) analytical package. The results were presented as counts, percentages, table of frequencies, and mean ± SD for continuous variables. The significance was declared at *P* value less than 0.05 and presented using narrative texts and tables.

### 2.5. Ethical Issues

#### 2.5.1. Ethical Approval

Ethical clearance was obtained from the ethical review committees, Colombo South Teaching Hospital, Kalubowila, and Faculty of Medical Sciences, University of Sri Jayewardenepura, Sri Lanka, and was approved by the Board of Study, Postgraduate Institute of Medicine, University of Colombo. Informed written consent was obtained from all participants.

## 3. Results

### 3.1. Sociodemographic Pattern and Risk Factors of Respondents

The total sample consisted of 384 hypertensive patients with a mean age of 59.32 (±12.34SD). This included 180 (46.9%) males and 204 (53.1%) females. The male : female ratio was approximately 9 : 10. The majority of patients were Sinhalese in ethnicity (84.1%). Most of patients were with primary or ordinary level educational status (65.9%), normal body mass index (54.9%), mild elevation of LDL cholesterol (76.3%), and coexistent ischemic heart disease (39.6%) (Table 1).

### 3.2. Knowledge about Hypertension among Respondents

The mean response values of overall questions related to knowledge about hypertension among majority of patients was reasonable (72.3%). However, 31% (95% CI 1.92-2.09) patients were aware about the normal values of blood pressure, and only 9.9% (95% CI 2.26-2.40) patients were aware about the cutoff values of hypertension. 69% (95% CI 1.64-1.88) patients had thought that the blood pressure increased with age. 41.7% (95% CI 1.67-1.81) patients believed that medication alone is not sufficient to control blood pressure. Most patients were aware that uncontrolled blood pressure could lead to heart failure (71.9%, 95% CI 1.42-1.58), heart attack (88.3%, 95% CI 1.15-1.27), and stroke (77.1%, 95% CI 1.33-1.49), whilst fewer patients were aware of renal (55.2%, 95% CI 1.69-1.88) and ophthalmological (57%, 95% CI 1.61-1.79) complications. Most of patients had adequate knowledge about the risk factors for developing of hypertension (Table 2).

### 3.3. Awareness about Hypertension among Respondents

The patients were interviewed about awareness of hypertension with pretested validated questionnaires and results were shown in Table 3. 81.5% (95% CI 1.18-1.28) patients were unaware about their hypertensive diagnosis. However, 24.7% (95% CI 2.09–2.25) patients could recall their blood pressure values at the time of diagnosis and 18.2% (95% CI 2.25–2.41) patients were aware of their target values of blood pressure. 29.7% (95% CI 2.0-2.17) patients could recall the blood pressure values of last visit. 41.9% patients misbelieve that their blood pressure was normal or low on last visit. Most patients knew that hypertension is a serious medical issue. 97.6% (95% CI 1.40-1.51) patients were aware of taking their medication important to control their blood pressure. 68.2% (95% CI 1.37-1.51) patients believed that adequate control of their blood pressure reduces complications. 70.3% (95% CI 1.31-1.43) patients had awareness of their target organ damage due to hypertension (Table 3).

### 3.4. Medication Adherence of Hypertension among Respondents

All participants were interviewed about adherence to their medications and reasons for nonadherence. The questionnaire contains six questions, which had yes, no, and cannot remember type responses and one multiple choice question. The results are shown in Table 4. The reasons for nonadherence were asked among patients through eight universal reasons, and the responses are shown in Table 5. Most of the patients had reasonable good drug compliance. The forgetfulness (69%, 95% of CI 1.26-1.36) was the common reason for nonadherence of their medication. The impact of side effects and misbeliefs on compliance was minimal. The cost and availability of drugs at state hospital were not a problem among them.

The patient's knowledge of risk factors and complications related to hypertension did not differ significantly with age, sex, and educational status (*P* > 0.05). However, their awareness of diagnosis, diagnostic values, cutoff values, and target values of hypertension differed significantly with the educational status (*P* < 0.05).

## 4. Discussion

The previous studies and our study have showed the similar findings indicating that although patients had good knowledge of hypertension, control of blood pressure remained suboptimal [5–7]. Moreover, in our study, most patients were unaware about the normal values of blood pressure (69%) and the cutoff values of hypertension (90.9%). Most of patients (81.5%) were aware about their hypertensive diagnosis. Furthermore, a large study by the Australian Heart Foundation in 1978 among hypertensive population showed that decreased level of knowledge and awareness with long term follow-up was 49% in 1978, 34% in 1993, and remained at 34% in 1998 [8]. Therefore, we think about patient's practices and attitude towards hypertension which had a significant positive impact on BP control rather than knowledge.

The results were highlighted in a previous study in Northern Sri Lanka which showed that 40.5% of patients were unaware of hypertensive status even though 69.5% had adequate knowledge about hypertension [9]. Another study among 424 hypertensive patients in Eastern Sri Lanka also showed that 92% of people had inadequate knowledge on the disease, its complications, and management strategies of hypertension with a overall knowledge score of <50% [10] which is in contrast to our findings. Another study among 525 hypertensive patients in 3 different healthcare systems over a 1-year period showed a significant association between BP control and knowledge about normal BP [11]. However, our study showed that knowledge about the risk factors for development of hypertension was adequate. Furthermore, patients were aware more about cardiac and neurological complications than renal and ophthalmological consequences in our study.

Recent research reports showed that hypertension knowledge is related to systolic blood pressure (SBP) control, and systolic blood pressure is a strong independent risk factor for cardiovascular mortality and morbidity [11]. However, critical elements of BP knowledge have not been adequately assessed or there is lack of data on whether patients understand the importance of their BP level, especially with regard to the systolic component of BP [12]. Recently, lack of knowledge of target systolic BP levels was shown to be an independent predictor of poor BP control. Therefore, assessment of awareness is importance in controlling their SBP. Most importantly, few patients could recall their blood pressure values at the time of diagnosis (24.7%) and were aware of their target values of blood pressure (18.2%) and could recall the blood pressure values of last visit (29.7%). Moreover, misbelief about blood pressure as either normal or low on the last visit was noted among them (41.9%). Patients who were unaware of their blood pressure thought that physicians did not emphasize the significance of blood pressure levels at clinic. These results strongly suggest that education of patients by health professionals at clinic visits was related to the importance of elevated blood pressure and cardiovascular risk. A recent study from Canada found a positive impact of BP tracker and a patient education on hypertension knowledge at clinic visits [13]. Therefore, the access to patients' clinic blood pressure data and perception of factors should be measured and evaluated at clinic visits. It is important to assess the extent of awareness of control of blood pressure levels among patients at clinics. That will be helpful to be part of educational programs and interventions designed to improve the control of hypertension. Furthermore, another study showed an improvement in both BP control and drug adherence, whilst patients were educated to measure their own BP and chart it, along with their drug-taking schedule as well [14].

Prescriptions of the multiple drugs and poor compliance of the patients are major challenges in clinical practices that results in failure of treatment of hypertension. Hypertension is mostly a chronic asymptomatic condition. Therefore, patients may not feel any physical symptoms and may forget to take their medicine or feel that there is no need to take them until they know the need to take drugs regularly for a long time. Our study showed that most patients have known that hypertension is a serious medical issue and were aware of taking their medication important to control their blood pressure with reasonable drug compliance. Furthermore, our research showed various reasons for patients not taking antihypertensive drugs. Among them, forgetfulness was common reason for nonadherence of their medication. Some people thought taking drugs was unnecessary as they were not experiencing symptoms of hypertension. Some of them preferred traditional medicines and have not taken due to religious misbeliefs. An article which reviewed adherence to cardiovascular medications from among 76 studies showed same findings. The poor knowledge, negative perception about medication, side effects, and high medication cost were the most common predictors of poor drug adherence [15]. Socioeconomic factors of drug nonadherence, such as medication cost and lack of availability of drugs, were also essential factors for drug adherence which was also observed in our study. Furthermore, drug adherence could be improved by enhancing access to drugs by sustainable financing, affordable prices, and reliable supply systems [16].

Lifestyle interventions have the potential to reduce the need or number of medications in treatment of hypertension which includes reduced alcohol intake, reduced sodium chloride intake, increased physical activity, and control of overweight. In addition, several studies have showed globally that demographic factors increase the risk of uncontrolled hypertension among hypertensive patients [17, 18]. Our results showed that the majority of patients were overweight and had high cholesterol level and sedentary life style. Moreover, our study demonstrated significant associations of hypertension control status with demographic characteristics and lifestyle factors such as smoking and alcohol drinking. Even though most patients were aware of risk factors of hypertension in our study, practicing life style intervention was minimal among them. Regular physical activities reduce BP through decreased body weight or favorable changes in body fat distribution. These findings also point out that the importance of having health education concerning physical activity and maintenance of normal body weight for patients with high BP [19]. Furthermore, individualized evaluation of perception, attitudes, beliefs, and outcome expectations is crucial role to understand observed behavioral change.

According to Farquhar's model of behavior change, our study results showed most patients had sufficient knowledge about hypertension, but only a few showed real motivations to change [20]. Moreover, few patients have interested in a behavioral change during conversation with them to control their hypertension in future. We also had opportunity to educate their hypertensive status in order to improve their awareness of hypertension at the end of the study. Moreover, physicians and healthcare professionals have identified mass media programs, printing materials, and video conferences as major sources to improve their knowledge and awareness of hypertension. Control of SBP and improved compliance should be achieved through an educational program that at least leads a concept of “knowing high BP” [21]. This recent research portrays the need to improve knowledge and awareness of hypertension in order to improve the medication adherence and optimum blood pressure control.

## 5. Conclusions and Recommendations

Our study revealed that the patients of a tertiary care hospital had sufficient general knowledge of hypertension but were unaware about their disease status and their diagnosis, target and recent blood pressure values, and about their target organ damage. Most of patients had adequate knowledge about the risk factors and complications of hypertension. Most of the patients had reasonable good drug compliance. The forgetfulness was commonly attributed for nonadherence. It is important to assess the extent of awareness of control of blood pressure levels among patients. That will be helpful to be part of educational programs and interventions designed to improve the control of hypertension. Therefore, healthcare professionals should implement individualized educational programmes to increase the awareness of disease status, appropriate blood pressure levels and adherence of treatment to improve outcome of patients. This study specifies potential areas where appropriate education of not only hypertension but also BP control could be an apt tool for the improvement of hypertension knowledge of patients.

### 5.1. Limitation of Study

We have not carried out a study to assess the knowledge, awareness, and medication adherence following the educational interventions. Therefore, further large-scale studies should be carried out to assess the participants' perceptional level following interventions.

## Figures and Tables

**Table 1:** The selected sociodemographic characteristics and risk factors among hypertensive patients.

Selected variables		Numbers	Percentage	95% confidence interval
Sex	Male	180	46.9	1.48–1.58
	Female	204	53.1	

Age (years)	20–29	8	2.1	4.40–4.66
	30–39	15	3.9	
	40–49	50	13.0	
	50–59	114	29.7	
	60–69	105	27.3	
	70–79	75	19.5	
	80–89	16	4.2	
	>90	1	0.3	

Ethnicity	Sinhala	323	84.1	1.18–1.29
	Tamil	34	8.9	
	Muslim	25	6.5	
	Others	2	0.5	

Educational level	No schooling	19	4.9	2.90–3.10
	Primary level	103	26.8	
	Ordinary level	150	39.1	
	Advanced level	90	23.4	
	Undergraduate level	14	3.6	
	Postgraduate level	8	2.1	

Occupation	Housewife	151	39.3	2.70–3.14
	Unemployed	46	12.0	
	Self-employed	91	23.7	
	Graduate student	4	1.0	
	Business	30	7.8	
	Officers	21	5.5	
	Professionals	16	4.2	
	Retired officers	23	6.0	
	Others	2	0.5	

Monthly income (Sri Lanka rupees)	Rs < 14,999	94	24.5	2.63–2.99
	Rs 15,000–29,999	108	28.1	
	Rs 30,000–44,999	82	21.4	
	Rs 45,000–59,999	45	11.7	
	Rs 60,000–74,999	22	5.7	
	Rs 75,000–89,999	12	3.1	
	Rs > 90,000	7	1.8	
	No income	14	3.6	

Smoking status	Never smoked	278	72.4	1.41–1.63
	Ex smoker	63	16.4	
	Current smoker	17	4.4	
	1–3 days/week	11	2.9	
	4–6 days/week	6	1.6	
	Everyday	9	2.3	

Alcoholic status	Never took alcohol	275	71.6	1.32–1.48
	Ex alcoholic	86	22.4	
	Current alcoholic	8	2.1	
	1–3 days/week	9	2.3	
	4–6 days/week	5	1.3	
	Everyday	1	0.3	

Body Mass Index (Kg/m^2^)	Underweight	10	2.6	2.40–2.53
	Normal	211	54.9	
	Overweight	137	35.7	
	Obese	26	6.8	

Serum LDL cholesterol (mg/dL)	<100	147	38.3	1.89–2.12
	100–149	146	38.0	
	150–199	64	16.7	
	>200	1	0.3	
	Not available	26	6.8	

Comorbidities	IHD	152	39.6	3.97–4.55
	Stroke	7	1.8	
	CKD	23	6.0	
	IHD and CKD	24	6.3	
	IHD and stroke	5	1.3	
	IHD, CKD, and stroke	4	1.0	
	None	169	44.1	

**Table 2:** The patient's knowledge on hypertension.

Questions	Response		Number	Percentage	95% CI
Knowing normal values of BP	Correctly known		119	31.0	1.92–2.09
	Wrongly known		41	10.7	
	Not known		224	58.3	
Knowing the cutoff values of HT	Correctly known		38	9.9	2.26–2.40
	Wrongly known		54	14.1	
	Not known		292	76.0	
BP correlation with age	Increased		265	69	1.64–1.88
	Decreased		22	5.7	
	No effect		22	5.7	
	Not known		75	19.5	
BP correlation with sex	Common among male		104	27.1	2.49–2.73
	Common among female		75	19.5	
	Equal among both sex		73	19.0	
	Not known		132	34.4	
Control of BP with medication alone	Sufficient		162	42.2	1.67–1.81
	Insufficient		160	41.7	
	Not known		62	16.1	

Knowledge about complications of HT	Yes		No	Not known	95%CI

Cerebral edema	163 (42.4%)		82 (21.4%)	139 (36.2%)	1.85–2.03
Heart failure	279 (71.9%)		24 (6.3%)	84 (21.9%)	1.42–1.58
Myocardial infarction	339 (88.3%)		8 (2.1%)	37 (9.6%)	1.15–1.27
Sudden visual loss	219 (57.0%)		61 (15.9%)	104 (27.1%)	1.61–1.79
Acute kidney injury	212 (55.2%)		43 (11.2%)	129 (33.6%)	1.69–1.88
Acute stroke	296 (77.1%)		21 (5.5%)	67 (17.4%)	1.33–1.49
Intracranial bleeding	223 (58.1%)		46 (12.0%)	109 (28.4%)	1.64–1.83

Knowledge about risk factors of HT	Increased	Decreased	No effect	Not known	95%CI

Smoking	279 (71.9%)	13 (3.2%)	12 (3.1%)	84 (21.9%)	1.33–1.54
Excess alcohol consumption	319 (83.1%)	1 (0.3%)	14 (3.6%)	50 (13.0%)	1.36–1.57
Excess spicy food consumption	189 (49.2%)	6 (1.6%)	71 (18.5%)	118 (30.7)	2.17–2.44
Excess fatty meals consumption	304 (79.2%)	3 (0.8%)	20 (5.2%)	57 (14.8%)	1.45–1.67
Excess salt consumption	308 ((80.2%)	5 (1.3%)	18 (4.7%)	53 (13.8%)	1.41–1.63
Lack of physical activity	302 (78.6%)	14 (3.6%)	17 (4.4%)	51 (13.3%)	1.43–1.65
Overweight	308 (80.2%)	2 (0.5%)	18 (4.7%)	56 (14.6%)	1.60–1.85
Family history	278 (72.4%)	3 (0.8%)	34 (8.9%)	69 (18.0%)	1.47–1.70
Aging	303 (78.9%)	4 (1.0%)	12 (3.1%)	65 (16.9%)	1.65–1.70

**Table 3:** The patient's awareness on hypertension.

Questions	Response	Numbers	Percentage	95%CI
Knowing about diagnosis of HT	Well known	313	81.5	1.18–1.28
	Unsure	53	13.8	
	Not known	18	4.7	
Knowing the values of BP at diagnosis	Correctly known	95	24.7	2.09–2.25
	Wrongly known	130	33.9	
	Not known	159	41.4	
Knowing target values of BP	Correctly known	70	18.2	2.25–2.41
	Wrongly known	117	30.5	
	Not known	197	51.3	
Knowing BP values of last clinic visit	Correctly known	114	29.7	2.0–2.17
	Wrongly known	127	33.1	
	Not known	143	37.2	
Seriousness of high BP	Very serious	165	42.9	1.61–1.74
	Serious	183	47.7	
	Not serious	36	9.4	
Importance of medication to control BP	Very important	217	56.5	1.40–1.51
	Important	158	41.1	
	Not important	9	2.3	
Good BP control reducing complications	Well known	262	68.2	1.37–1.51
	Unsure	73	19.0	
	Not known	49	12.8	
HT leading to target organ damage	Well known	270	70.3	1.31–1.43
	Unsure	85	22.1	
	Not known	29	7.6	

Target organ damage	Well known	Unsure	Not known	95% CI

Kidney	216 (56.3%)	71 (18.5%)	97 (25.3%)	1.60–1.78
Heart	327 (85.2%)	30 (7.8%)	30 (7.8%)	1.16–1.27
Brain	255 (66.4%)	76 (19.8%)	76 (19.8%)	1.40–1.55
Eye	215 (56.0)	86 (22.4%)	83 (21.6%)	1.57–1.74

**Table 4:** The patient's medication adherence of hypertension.

Questions	Yes	Response No	Can't remember	95%CI
Taking drugs regularly till last month	238 (62%)	101 (26.3%)	45 (11.7%)	1.45–1.59
Taking drugs regularly for last 2 weeks	278 (72.4%)	75 (19.5%)	31 (8.1%)	1.32–1.46
Taking drugs regularly yesterday	303 (78.9%)	68 (17.7%)	13 (3.4%)	1.22–1.33
Stopping drugs due to side effects	55 (14.3%)	300 (78.1%)	29 (7.6%)	1.90–2.0
Taking drugs outside home stay	212 (55.2%)	106 (27.6%)	66 (17.2%)	1.80–1.91
Stopping drugs due to normal BP level	85 (22.1%)	272 (70.8%)	27 (7.0%)	1.56–1.72

Forgetting to take drugs in the past	Numbers	Percentage	95% CI	

Never	178	46.4	1.77–1.96	
Rarely	99	25.8		
Sometimes	88	22.9		
Frequently	19	4.9		

**Table 5:** The reasons for nonadherence of medication among hypertensive patients.

Reasons	Numbers	Percentage	95%CI
Poor knowledge of HT	77	20.1	1.77–1.86
Poor knowledge of medication	68	17.7	1.79–1.86
Religious beliefs	34	8.9	1.88–1.94
Lack of belief on medication	56	14.6	1.82–1.89
Side effects of medication	61	15.9	1.80–1.88
Forgetfulness	265	69.0	1.26–1.36
Out of supply of medication	51	13.3	1.83–1.90
Cost of medication	39	10.2	1.87–1.93

## Data Availability

The data used to support the findings of this study are available from the corresponding author upon request.

## Abbreviations

BP: Blood pressure
CI: Confidence interval
CKD: Chronic kidney disease
HT: Hypertension
IHD: Ischemic heart disease
LDL: Low-density cholesterol
SBP: Systolic blood pressure
SD: Standard deviation.
